# Psychosocial Concomitants of Infertility: A Narrative Review

**DOI:** 10.7759/cureus.80250

**Published:** 2025-03-08

**Authors:** Charu Jain, Waheeda Khan

**Affiliations:** 1 Faculty of Social and Behavioural Sciences, Shree Guru Gobind Singh Tricentenary (SGT) University, Gurgaon, IND

**Keywords:** anxiety, family cohesion, infertility, narrative review, partner dynamics, psychological factors, quality of life, social factors

## Abstract

Infertility is a distressing and challenging condition that poses a significant social and public health concern. Among individuals of reproductive age, awareness of the factors associated with infertility is crucial. The prevalence of infertility has evolved over time, with distinct trends observable both globally and within India.

The primary objective of the study was to highlight the psychological and social factors associated with infertility. A comprehensive literature search was conducted to examine the psychological and social factors related to infertility. The electronic databases utilized for this search included PubMed and Google Scholar. The findings from the selected studies were synthesized into a narrative review. A total of 46 studies investigating the concomitants of infertility were analyzed. Among them, 32 studies focused on psychological aspects, while 14 addressed social factors.

The psychological aspects covered in the studies included quality of life (QoL), anxiety and depression, gender differences, psychiatric disorders, coping mechanisms, and subjective well-being. The social factors examined included partner dynamics - further categorized into sociodemographic influences, marital adjustment, and violence against women - family cohesion, lack of awareness, and socio-environmental influences.

This review provides an overview of the psychological and social dimensions of infertility, while also identifying gaps in existing research. It underscores the need for further studies, particularly focusing on rural populations, increasing awareness among men, and understanding the impact of infertility-related violence. Ultimately, the research highlights the complexity of infertility and emphasizes the importance of continued research and intervention efforts to address its multifaceted implications.

## Introduction and background

Fertility is a core aspect of human life, and the emotional turmoil of infertility stands as one of its most profound challenges. Infertility affects millions of individuals globally, and across cultures, childlessness is often viewed as an undesirable social role and an *unexpected life transition*. The World Health Organization (WHO) defines infertility as a disease of the reproductive system characterized by the failure to achieve pregnancy after 12 or more months of regular, unprotected intercourse [1].

For many women, infertility and involuntary childlessness represent a chronic life crisis that can profoundly impact later life transitions [2]. The emotional toll of infertility is substantial, with involuntary childlessness significantly affecting the well-being of infertile couples. Many women describe infertility as the most serious emotional crisis they have ever faced [3]. The psychological impact includes increased stress, anxiety, depression, and lower self-esteem. The emotional burden can also strain marital relationships, leading to communication difficulties and reduced intimacy. Men and women often experience and process infertility differently, with women frequently reporting greater emotional distress. The persistent uncertainty and repeated disappointments associated with infertility treatments can further exacerbate psychological distress, making infertility a deeply challenging experience.

Beyond being a critical public health issue, infertility carries serious social consequences, particularly in developing countries, where parenthood is highly valued. Societal structures often necessitate children for the care and maintenance of aging parents. This expectation is present not only in underprivileged communities but also in societies with established social support systems, where children are still expected to provide care for the elderly. Given these psychological, familial, and societal pressures to conceive, infertility can be particularly distressing for couples, especially women, in such settings [4]. Women, in particular, may face stigma, social exclusion, and even marital instability due to their inability to conceive.

The objective of this review is to explore the psychological and social factors associated with infertility. By synthesizing existing research, this review seeks to provide a comprehensive understanding of the multifaceted concomitants of infertility and highlight areas that require further attention in clinical practice and future research.

## Review

Method of literature search

**Figure 1:**
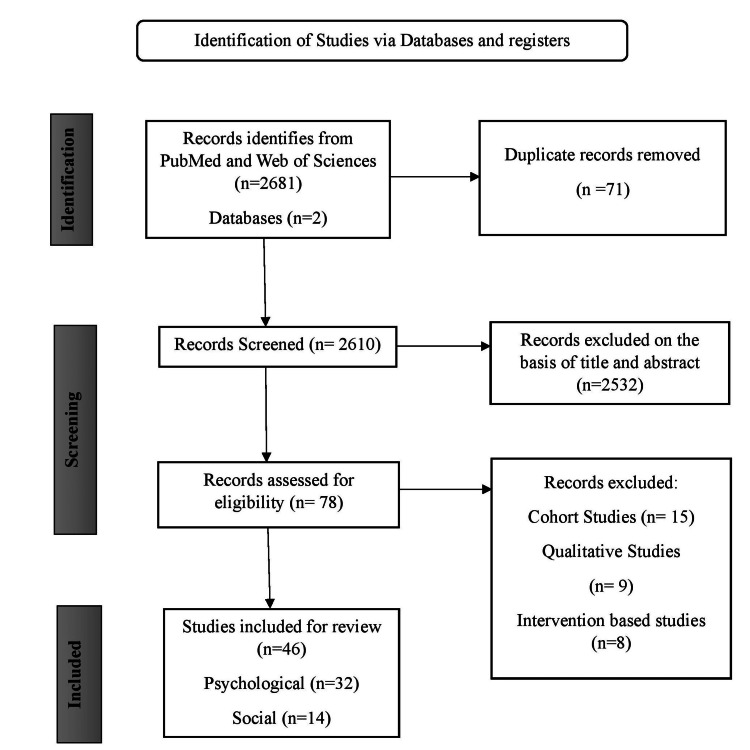
Flow diagram describing the selection process for relevant research studies.

A literature search was planned to explore the psychological and social factors of infertility to conduct the narrative review. The search engines used to conduct the electronic literature searches were PubMed and Web of Sciences (Figure 1).

A combination of keywords was used to make the searches such as (“Behavioural factors” OR “Lifestyle factors” OR “Alcohol” OR “smoking” OR “caffeine” OR “exercise” OR “diet” OR “exposure to pollutants” OR “sexual activity”) AND (“Psychological factors” OR “Attachment styles” OR “defense style” OR “coping style” OR “anxiety” OR “feelings of anxiousness” OR “depression” OR “hopeful of success” OR “motivation” OR “stress” OR “shame” OR “guilt” OR “self- esteem” OR “psychological trauma” OR “sleep disturbances” OR “eating disorders” OR (“Social factors” OR “Marital quality” OR “support” OR “awareness of condition” OR “society”s attitude” OR “religious beliefs” OR “role of doctors” OR “education” OR “urbanization” OR “occupational distribution” OR “developing nation” OR “developed nation” OR “status of women”) AND (Infertility OR “Infertile Couple” OR “Infertile Women” OR Fertility OR “Fertile Couple” OR “Fertile Women”). Search results were limited according to the inclusion and exclusion criteria (Table 1). The findings of these studies were synthesized into a narrative review.

**Table 1:** Study-specific inclusion and exclusion criteria.

Category	Criteria for inclusion	Criteria for exclusion
Language	English	All non-English literature
Years of publications	2000-2024	NA
Study design	Cross-sectional studies, observational studies, case-control studies	Individual randomized controlled trials, quasi-experimental trials, non-randomized trials, intervention-based study, experimental studies
Publications	Complete full-text articles	Systematic reviews, meta-analyses, protocols, stand-alone abstracts, studies lacking original/experimental data, narrative reviews, editorials, letters to the editors, and similar publications
Populations	Married adults (25-40 years of age), diagnosed with infertility, and never been for treatment before the study	Unmarried adults (<25 years) and children
Gender	Both male and female	Transgender, LGBTQIA+
Geographical setting	Any country	NA
Others	Access to full-text articles	Inability to retrieve the full-text article

Results

A total of 2,681 articles were initially retrieved from searches across two databases. After removing duplicates, 2,610 articles remained and were screened based on the relevance of their titles and abstracts to the study objectives. Following this screening, 78 articles were deemed eligible for full-text review. Of these, 32 were subsequently excluded based on the inclusion criteria for the review (Figure 1). 

Psychological Concomitants of Infertility

Infertility can lead to significant psychological distress, affecting both individuals and couples as they navigate the emotional and social challenges associated with their condition [5]. The psychological impact of infertility is often compounded by the prolonged diagnostic processes and treatments, which can be physically and emotionally exhausting. These stressors may disrupt emotional well-being, contribute to anxiety and depression, and interfere with various aspects of daily life, including intimate relationships. In many cases, the emotional strain of infertility can negatively affect sexual functioning, as feelings of frustration, guilt, and inadequacy take a toll on both partners, further complicating their journey toward parenthood [6].

Quality of Life

The WHO defines quality of life (QoL) as an individual’s perception of their position in life within the context of their cultural and value systems, as well as in relation to their goals, expectations, standards, and concerns. In the literature, a more specific definition has been introduced for fertility-related QoL (FertiQoL), which assesses the impact of fertility issues across various life dimensions. Infertility-related QoL categorizes it as a social disease [5], affecting approximately 10%-18% of married couples worldwide. Research indicates that infertility negatively impacts QoL and sexual functioning in both men and women. Additionally, women over the age of 30, those with lower education levels, and those who are occupationally inactive have been identified as being at higher risk [6-7]. A study conducted in Turkey found that women’s QoL - specifically in the domains of emotional well-being, mind/body connection, core functioning, and tolerability - was more adversely affected than that of their husbands [8]. Conversely, another study reported that women living in rural areas experienced lower QoL across economic, emotional, sexual, physical, and psychological dimensions compared to infertile women [5,9].

Anxiety and Depression

A total of eight studies have extensively examined anxiety and depression within the context of infertility. Anxiety is often linked to apprehension about the future, whereas depression is associated with feelings of hopelessness and helplessness. Cross-sectional studies conducted in Hungary [10], Poland [11], China [12], Pakistan [13], Ghana [14], New York [15], and Japan [16] consistently indicate that infertile women experience significantly higher levels of anxiety and depressive symptoms compared to their fertile counterparts, reflecting a poorer psychological state among infertile couples. Anxiety symptoms in infertile couples may arise due to various factors, including unknown etiology, uncertainty regarding prognosis and treatment outcomes, prolonged waiting periods, financial stress associated with treatment and counseling, and societal or cultural pressures [17]. In contrast, depressive symptoms have been significantly associated with factors such as social and sexual concerns [10], older age, rural residence, lower household income, lower education level, adverse life events, and prolonged infertility duration [12].

Gender Differences

In Middle Eastern countries such as Iran, fatherhood is traditionally equated with manhood, and infertile men often face stigma, being perceived as futile and having their masculinity questioned [18]. Similarly, female infertility is stigmatized across both Western and non-Western cultures. However, in non-Western societies, the pressure is particularly severe due to the cultural emphasis on childbearing as a defining aspect of womanhood, the high value placed on children by extended families, and the challenges associated with legal or permanent adoption [19]. Psychological distress in infertile men has been linked to increased cigarette and alcohol consumption, with depressed men showing higher smoking rates compared to the general population [18,20]. While both genders experience psychological distress, studies indicate that women report a higher number of depressive symptoms than men [21]. Among women with primary infertility, lower educational status, poor marital communication, high perceived importance of having children, and a history of infertility evaluation or treatment were independently associated with increased psychological distress [22]. Additionally, ethnicity played a significant role in women's depression, although this relationship was not observed in men [21]. In contrast, a higher level of education and diabetes was associated with lower self-esteem scores [23], whereas greater self-esteem, body esteem, and emotional self-efficacy were correlated with improved psychological well-being [24-25].

Coping

Coping styles are common responses to stress, and the type of coping strategy employed plays a crucial role in stress management. Adaptive coping strategies, such as task- or problem-focused approaches, contribute to better stress adaptation, whereas emotion-focused strategies are associated with increased distress. While no significant gender differences in coping styles were observed, emotional coping showed a strong correlation with psychological distress [26]. However, another study found that women, in comparison to men, reported significantly greater use of coping strategies such as self-blame, avoidance, information and emotional support seeking, and cognitive restructuring in response to infertility [27]. Perceptions of infertility also varied based on education level, with less educated women attributing their infertility to supernatural causes, such as evil spirits or witchcraft affecting menstruation, whereas more educated women associated it with nutritional, lifestyle, marital, and psychosexual factors [28]. Additionally, lower levels of infertility-related distress were linked to a more positive emotional reaction upon receiving an infertility diagnosis [29].

Psychiatric Disorders

Several studies have examined the prevalence of psychiatric disorders in the context of infertility. Psychiatric disorders encompass a wide range of conditions that affect mood, emotions, and behavior. The psychological impact of infertility has been explored at multiple levels, including the concurrence of psychiatric disorders and associated risk factors in infertile couples [30]. Both organically and functionally infertile couples reported heightened irritability, resentment, and hostility, often accompanied by feelings of inadequacy and inferiority, particularly when comparing themselves to others [31]. Women were found to experience more somatic complaints than men. Both infertile men and women exhibited a higher likelihood of psychiatric morbidity compared to their fertile counterparts; however, statistical significance was observed only for dysthymia and anxiety disorders in infertile childless women and panic disorder in infertile women with a child [32]. Additionally, significant gender differences were observed in the prevalence of psychiatric disorders, with notable disparities in bipolar and substance-related disorders. Among women diagnosed with psychiatric disorders, financial stress and having a polygamous husband were significantly correlated with their condition [30]. Furthermore, male partners of infertile couples exhibited significantly higher acute psychological symptoms compared to those of pregnant women [33].

Subjective Well-Being

Subjective well-being in the context of infertility has been evaluated through factors such as general health and life meaning, providing insight into an individual’s overall psychological status. Women tend to experience infertility-related strain more explicitly than men, with those facing unwanted childlessness displaying higher levels of femininity compared to the reference population - possibly as a compensatory response to affirm their womanhood despite their inability to conceive. Hungarian men, in comparison to a Canadian reference population, generally perceive infertility as more stressful and exhibit a stronger need for parenthood. Additionally, Hungarian infertile men and women demonstrated a greater level of marital adjustment [34].

Social Concomitants of Infertility

The experience of infertility is deeply influenced by the sociocultural environment, which shapes individual perceptions, expectations, and coping mechanisms. In collectivist societies, where family and societal expectations play a crucial role, infertility is not merely a personal struggle but a shared concern that affects the entire family unit. Within these cultural frameworks, family members are interconnected not only through biological ties but also through social and psychological relationships, creating added pressure on individuals facing infertility [35]. The societal emphasis on parenthood as a fundamental milestone can lead to heightened emotional distress, stigma, and even strained interpersonal relationships, further complicating the psychological burden of infertility.

Partner Dynamics in Infertility

Infertility affects various aspects of a couple’s relationship, including emotional well-being, marital satisfaction, and social interactions. The psychological and social consequences of infertility often influence partner dynamics, shaping how couples cope with stress, communicate, and support each other throughout the journey. Several factors, including sociodemographics, psychological distress, and the prevalence of intimate partner violence, play a role in shaping these dynamics.

Sociodemographic Influences on Partner Dynamics

Parenthood constitutes a significant stage in the psychosexual and social development of both men and women, allowing individuals to achieve life goals and fulfill personal needs. Infertile couples often engage in diagnostic and therapeutic processes in search of a solution to what they perceive as a major life problem [36]. Research indicates that individuals aged 26-30 years report lower psychological distress compared to other age groups [35]. Psychological distress, particularly severe depressive symptoms, has been significantly linked to higher levels of infertility-related distress at both the individual and partner levels [37]. Socioeconomic factors, including age, education level, and financial stability, have been considered critical variables influencing the degree of anxiety, depression, and anger among infertile couples. Additional factors such as cultural variations, infertility duration, and whether infertility is attributed to men or women also contribute to emotional and relational stress [38].

Marital Adjustment and Emotional Support

Infertility-related stress has been found to impact marital relationships in contrasting ways - either exacerbating conflicts or fostering deeper trust and intimacy [36]. In Japan, a lack of emotional support from husbands has been identified as a key source of anxiety and depression among infertile women [39]. Women frequently express frustration over their partners' perceived emotional detachment and lack of communication regarding infertility [40]. Research also suggests that incongruence in marital satisfaction, particularly differences in attitudes toward parenthood and child-free living, negatively influences marital adjustment [41]. However, opposing findings indicate that infertile couples may have a lower likelihood of experiencing significant marital interaction disorders compared to the general population.

Psychological distress is more pronounced among women, who report higher levels of anxiety, depression, and somatization in response to infertility. While men exhibit only a slightly higher tendency for somatization, women tend to experience more severe stress related to their infertility status [42].

Violence Against Women in the Context of Infertility

The intersection of infertility and intimate partner violence is a critical issue, with research showing that women with infertility often fear losing their spouses' interest. Employment status and the duration of infertility significantly influence the risk of experiencing abuse. Women solely dependent on their husbands for financial support are more likely to experience violence, although this association may be masked by other factors [43].

A significant proportion of infertile women (64%) report experiencing some form of abuse - whether verbal or physical - due to their infertility [19]. The increased prevalence of sexual assault among infertile women is another concerning aspect that cannot be overlooked [44]. These findings highlight the urgent need for interventions aimed at addressing domestic violence and providing emotional and psychological support to infertile women.

Family Support

Family serves as an individual’s sociocultural environment, significantly influencing health, mental well-being, and behavioral patterns. Family functioning, particularly in terms of adaptability and cohesion, plays a crucial role in shaping responses to stress. Family adaptability refers to the ability to adjust roles in response to challenges, while family cohesion represents the emotional bond between family members. Within the context of infertility, women in infertile couples reported higher dissatisfaction with family cohesion compared to men, indicating lower satisfaction with the emotional connection in their relationships. Additionally, women exhibited greater infertility-related global stress, experiencing heightened distress in areas such as social concerns, relationship concerns, the need for parenthood, and sexual concerns. In men, family cohesion and education level were negatively correlated with infertility-related stress, while in women, family adaptability and education level showed a similar negative association with stress [45].

Lack of Awareness

Insufficient knowledge of factors associated with infertility could lead some couples to engage, limited knowledge of factors associated with infertility may lead couples to unknowingly engage in behaviors that reduce their chances of conceiving. A study focusing on men revealed that they could correctly identify only half of the risk factors and health issues related to male infertility. While many men recognized the impact of substance use (e.g., smoking) and health-related risks (e.g., sexually transmitted infections) on fertility, a significant minority (20%-40%) remained unaware of these associations. Less than half of the respondents were aware of the effects of common health conditions such as high cholesterol, obesity, and diabetes on male fertility. Additionally, awareness was even lower regarding the impact of daily activities and excessive heat exposure, such as laptop use and frequent hot tub use. While men demonstrated moderate awareness of certain fixed risk factors, such as cancer treatment, there was a notable gap in knowledge regarding male reproductive anatomy (e.g., testicle size, delayed puberty) and its link to infertility. Poor understanding of these fixed risks may lead to delays in diagnosis and treatment, whereas greater awareness could encourage early assessment and intervention. The study also highlighted that men were least informed about the health consequences of male infertility, including the increased risks of metabolic syndrome and cardiovascular disease. Furthermore, only about 50% of men identified depression because of infertility [46].

Socio-environmental Factors Affecting Infertility

The COVID-19 pandemic introduced unprecedented challenges, affecting various aspects of life, including fertility-related concerns. Sexual activity, an essential component of well-being and a key indicator of QoL has been significantly impacted by the crisis. Anxiety, lack of privacy, health-related fears, and psychosomatic symptoms induced by lockdown measures have contributed to a decline in sexual health, particularly among individuals struggling with infertility, who may be more vulnerable to stress and crises. The frequency of sexual activity, anxiety symptoms, changes in sexual satisfaction during lockdown, and income levels were all associated with the quality of couple relationships. Participants experiencing high-stress levels reported significant perceived changes in sexual behavior compared to those with lower stress levels. A notable decline in sexual satisfaction, sexual desire, and frequency of sexual activity was observed in individuals experiencing extreme stress. Female participants exhibited lower Quality of Marriage Index (QMI) scores than their male counterparts. Moreover, a stronger decline in sexual satisfaction, desire, and activity was reported among individuals with poor relationship quality compared to those in stable relationships. Anxiety symptoms emerged as a significant risk factor for deteriorating relationship quality and were found to negatively predict QMI scores [47].

Attitude Toward Treatment

Health behaviors and attitudes are the actions and beliefs that influence an individual's decisions regarding their well-being and the path they choose to follow in managing their health. A high percentage of participants emphasized the importance of a patient-centered approach in fertility clinics. However, fewer individuals showed interest in professional psychosocial services, and an even smaller number intended to utilize these services [48]. Notably, men were more likely to choose assisted reproduction treatment for their partners (79%) rather than for themselves (56%) [49].

Discussion

This research highlights the psychosocial aspects of infertility, identifying various psychological and social factors through a literature review. It has been seen that Infertility affects QoL and is associated with psychiatric disorders such as somatization, anxiety, and depression. Gender differences in psychological responses and coping mechanisms have also been observed, influencing subjective well-being. Beyond individual effects, social factors play a crucial role in infertility. Partner relationships, including sociodemographic influences, marital adjustment, and family cohesion, significantly impact psychological outcomes. Issues such as violence against infertile women and limited awareness among infertile men further contribute to the emotional burden. Additionally, socio-environmental factors, including external stressors like economic conditions and global crises such as COVID-19, have been linked to increased distress in infertile couples.

The lower QoL observed in women can be attributed to their compensatory roles in both financial and emotional aspects, which add to their burden while coping with infertility [5]. Infertility is often perceived as a deficiency, leading women to experience profound feelings of disappointment and insufficiency when they are unable to conceive [8]. Additionally, dissatisfaction in older women may stem from a less assertive approach toward infertility care services and a heightened vulnerability to the emotional distress of childlessness, making them more susceptible to the negative psychological effects of infertility [50].

The heightened levels of anxiety, depression, and psychiatric disorders in infertile individuals can be attributed to the differences in how partners approach infertility. Women tend to be more psychologically affected than their male partners, as they often feel a greater sense of responsibility for infertility and undergo more invasive medical treatments. Additionally, women are more likely to experience emotional distress, a loss of self-esteem, and a stronger need to express their emotions about their struggles with conception compared to men [11]. Men's greater psychological resilience may be explained by societal norms that grant them more rights, power, and authority in decision-making. In patriarchal and polygamous societies, infertile women are frequently stigmatized and blamed by their spouses and in-laws, leading to depression and significantly contributing to their lower QoL [13]. Furthermore, the high prevalence of somatization among infertile individuals may stem from difficulties in managing stress, expressing emotions, and communicating distress, often serving as a nonverbal cry for help [31].

Partner dynamics play a crucial role in how couples navigate infertility-related stress. A higher level of marital adjustment is observed when partners perceive themselves as capable of helping each other cope with stress and effectively communicating their emotional struggles [41]. Men's socially expected role as the protector of the family may drive them to adopt dyadic coping strategies, aiming to support their partners in managing infertility-related distress and shielding them from emotional suffering [40]. However, economic dependence can place women in a vulnerable position within their relationships. When a woman lacks financial independence, she becomes entirely reliant on her spouse for necessities such as food, shelter, and overall welfare. This dependence may increase her risk of experiencing domestic violence, which often goes unreported due to fears of stigma, reprisal, shame, and blame [43]. These factors contribute to the complexity of partner dynamics, influencing both marital adjustment and individual psychological well-being.

Lack of awareness and sociodemographic influences play a significant role in how individuals perceive and manage infertility. Men often rely on online sources for fertility-related information, which aligns with previous studies suggesting their preference for seeking medical knowledge on the Internet. However, online articles alone may not be an effective tool for long-term retention of fertility knowledge, potentially leading to misinformation or gaps in understanding [46].

Additionally, the quality of a couple's relationship was closely linked to their mental health during the COVID-19 pandemic. The stress and uncertainty brought by the pandemic may have exacerbated emotional distress among infertile couples, further impacting their psychological well-being and coping mechanisms [47]. This highlights the importance of both reliable education on fertility and strong interpersonal support systems in mitigating the emotional burden of infertility.

Attitude toward treatment is shaped by the significant stressors linked to infertility and its medical management. The psychological burden of infertility, combined with the challenges of undergoing multiple cycles of fertility treatment, is well-documented. These stressors not only affect mental health but also contribute to financial difficulties and strain relationships both at home and in the workplace. This accumulated stress may play a role in the adoption of unhealthy behaviors among women undergoing fertility treatments [48]. Interestingly, couples who perceived that the hardships of infertility had brought some positive changes to their marriage were the most inclined to seek psychosocial support services. This suggests that individuals who acknowledge the emotional toll of infertility may also be more receptive to interventions aimed at improving mental well-being and relationship dynamics [49].

Several limitations of this review should be considered when interpreting the findings. First, as a narrative review, it may not have encompassed all relevant studies in the field. However, the primary objective was to highlight the psychological and social concomitants of infertility, particularly across diverse countries and contexts. Second, partner dynamics were categorized into sociodemographic influences, marital adjustment and emotional support, and violence against women in the context of infertility; however, alternative classifications may also be valid.

## Conclusions

There is increasing evidence that infertility is emerging as a significant health concern, particularly given the lifestyle choices of today’s youth. This narrative review aimed to explore the psychological and social factors associated with infertility. Existing research has identified various psychological aspects linked to infertility, including QoL, anxiety, depression, psychiatric disorders, coping mechanisms, and subjective well-being. On the social front, key factors include partner dynamics, lack of awareness, attitudes toward treatment, and socio-environmental challenges related to infertility.

While this review provides a comprehensive understanding of infertility and its contributing factors, it also highlights the need for further research, particularly in rural populations, increasing awareness among men, and examining the impact of infertility-related violence. The biological factors associated with infertility to provide a more comprehensive understanding can also be reviewed. Ultimately, this review underscores the complexity of infertility and emphasizes the importance of continued efforts to address its psychological and social dimensions.
